# Mental health professionals’ attitudes towards patients with borderline personality disorder: The role of disgust

**DOI:** 10.1192/j.eurpsy.2022.947

**Published:** 2022-09-01

**Authors:** C. Papathanasiou, S. Stylianidis

**Affiliations:** Panteion University of Social and Political Sciences, Psychology, Athens, Greece

**Keywords:** borderline personality disorder, mental health professionals, disgust, attitudes

## Abstract

**Introduction:**

Mental health professionals’ derogatory attitudes towards patients diagnosed with borderline personality disorder (BPD) may negatively affect treatment outcomes.

**Objectives:**

We aimed to identify a) negative attitudes exhibited by mental health professionals towards BPD patients and b) the effects of disgust propensity and disgust sensitivity on theses negative attitudes.

**Methods:**

Mental health professionals (N = 136) completed questionnaires on attitudes towards BPD patients, disgust propensity/sensitivity, and sociodemographic variables.

**Results:**

Significant differences in negative attitudes toward BPD patients based on gender, marital status, occupational subgroup, educational level, psychotherapy training, level of exposure to BPD patients, and political ideology were found. Results suggested BPD patients are viewed by mental health professionals as ineffective, incomprehensible, dangerous, unworthy, immoral, undesirable to be with, and dissimilar to the mental health professionals. Moreover, disgust propensity and the pathogen component of disgust sensitivity were associated with stronger negative attitudes towards BPD patients.

**Conclusions:**

The findings emphasize the importance of mental health professionals’ awareness of the emotion of disgust as a relevant factor to their negative attitudes towards BPD patients.

**Disclosure:**

No significant relationships.

